# Role of dietary fiber and lifestyle modification in gut health and sleep quality

**DOI:** 10.3389/fnut.2024.1324793

**Published:** 2024-04-03

**Authors:** Amjad Ali Bacha, Muhammad Suhail, Fuad A. Awwad, Emad A. A. Ismail, Hijaz Ahmad

**Affiliations:** ^1^Department of Human Nutrition, The University of Agriculture Peshawar, Peshawar, Pakistan; ^2^Amir Muhammad Khan Campus Mardan, The University of Agriculture Peshawar, Peshawar, Pakistan; ^3^Department of Quantitative Analysis, College of Business Administration, King Saud University, Riyadh, Saudi Arabia; ^4^Center for Applied Mathematics and Bioinformatics, Gulf University for Science and Technology, Mishref, Kuwait; ^5^Department of Computer Science and Mathematics, Lebanese American University, Beirut, Lebanon; ^6^Section of Mathematics, International Telematic University Uninettuno, Rome, Italy; ^7^Near East University, Operational Research Center in Healthcare, Nicosia, Türkiye

**Keywords:** sleep analysis, gastrointestinal tract, PSQI, GIT score, psyllium husk fiber, lifestyle modification, dietary fiber

## Abstract

Dietary fiber has an immense role in the gut microbiome by modulating juvenile growth, immune system maturation, glucose, and lipid metabolism. Lifestyle changes might disrupt gut microbiota symbiosis, leading to various chronic diseases with underlying inflammatory conditions, obesity, and its associated pathologies. An interventional study of 16 weeks examined the impact of psyllium husk fiber with and without lifestyle modification on gut health and sleep quality in people with central obesity (men = 60 and women = 60), those aged from 40 to 60 years, those having WC ≥ 90 cm (men) and WC ≥ 80 cm (women), and no history of any chronic disease or regular medication. The participants were subgrouped into three intervention groups, namely, the psyllium husk fiber (PSH) group, the lifestyle modification (LSM) group, and the LSM&PSH group and control group with equal gender bifurcation (men = 15 and women = 15). A 24-h dietary recall, gastrointestinal tract (GIT) symptoms, and sleep quality analysis data were collected on validated questionnaires. The analyses of variance and covariance were used for baseline and post-intervention, respectively. Student's *t*-test was applied for pre- and post-intervention changes on the variable of interest. The intervention effect on GIT health was highly significant (*P* < 0.001). The mean GIT scores of the LSM, PSH, and LSM&PSH groups were 2.99 ± 0.14, 2.49 ± 0.14, and 2.71 ± 0.14, respectively, compared to the mean GIT scores of the control group. No significant (*P* = 0.205) effect of either intervention was observed on sleep quality. The study concluded that psyllium husk fiber significantly improved the GIT symptoms, while no significant effect of the intervention was observed on sleep quality analysis.

## 1 Introduction

Diet and nutrition are significant aspects in the promotion and maintaining of good health throughout one's life; their function as predictors of chronic non-communicable diseases is well-recognized, and they thus hold a major place in preventive medicine (1). The World Health Organization (2) reports that non-communicable diseases (NCDs) cause 38 million deaths annually. By 2020, the proportion of non-communicable diseases (NCDs) is expected to increase, contributing to 75% of all fatalities globally. Of these deaths, 71% will be attributable to ischemic heart disease (IHD), 75% to stroke, and 70% to diabetes in developing nations (3).

There is growing recognition of the role of diet and other environmental factors in modulating the composition and metabolic activity of the human gut microbiota, which in turn can impact health (4). Along the GI tract's length, there are variations in microbe quantity, kind, and function. The majority, however, are concentrated in the large intestine, where they support fecal bulk and ferment undigested food items, especially carbohydrates and fiber (5). There are accumulating symptoms that indicate that abnormalities in gut microbial populations are linked to diseases, especially inflammatory bowel disease (IBD) (6), and may serve as contributing factors. Some bioactive substances, such as vitamins, are useful, while others are poisonous and are produced by microorganisms in the gut (7). Along the intestine, host immune systems, including a mucus barrier, help prevent potentially hazardous germs from causing tissue damage. By competing for nutrition and colonization sites, a diverse and robust community of good gut bacteria helps keep dangerous bacteria at bay. It has been demonstrated that dietary fibers significantly affect the gut microbiota's functionality and composition, which has positive implications on health due to their structural, physical, and chemical properties such as viscosity, water binding and bulking ability, and fermentability (8). To sustain microbial richness with more apparent (additive or synergetic) impacts on the immunological status and metabolic health, mixing various fibers that stimulate a multitude of different bacterial species may be helpful (9). High-fiber diets benefit the host's health by influencing glucose and cholesterol metabolism, among other things. Important pathways include nutrition absorption control and SCFA synthesis (10). Ingestion of live beneficial bacteria (probiotics) may also contribute to health maintenance (8).

Short-chain fatty acids (SCFA), which are produced by large bowel bacteria from the fermentation of fiber and protein, are some of the most prevalent and physiologically significant products. Colorectal tissues and bacteria rely on SCFA for energy, as they are essential for the proper functioning of cellular mechanisms that ensure tissue integrity (11, 12). SCFA can enter the blood and influence immunological function and inflammation in the lungs and other tissues (13). Numerous additional products, such as *Bifidobacterium*, which produce specific vitamins in the large intestine, are noteworthy for their impact on health (e.g., K, B_12_, biotin, folate, and thiamine) (14). On the whole, the effect of non-dietary lifestyle factors on the gut microbiota has been neglected. As risk factors for colorectal cancer, smoking and a lack of exercise can have a major impact on the large intestine (and potentially the microbiome) (15). Obesity-related changes in microbial communities may be influenced by exercise (or, conversely, its absence). The diversity of gut microbial communities in professional athletes is a result of exercise and food (16).

All living things require sleep to maintain good mental health, facilitate learning, and remove metabolic waste from the brain (17). Homeostatic and circadian processes control sleep behavior; the latter seems to be influenced by the genetic makeup of the gut microbiota (18, 19). Epidemiological evidence has suggested that poor sleep health is associated with adverse outcomes such as cardiovascular diseases (20, 21), metabolic syndrome (MetS), and mental illnesses (22, 23) and plays a vital role in the development of MetS (24). Generally, sleep health has two main dimensions: duration and quality (25, 26). Sleep length and quality may overlap to some extent, but there are qualitative variations between both. In addition, previous research has demonstrated that associations between sleep length and sleep quality are weak (27), implying that the two distinct sleep estimation areas may have different health consequences (28, 29). Previous studies have shown that the average self-reported sleep duration has decreased from over 8 h in the 1960s to 6.5 h in 2012 (30, 31).

Physiochemical properties, including solubility, viscosity, and fermentability, control how dietary fiber behaves in the human gastrointestinal tract (32). Additionally, the quantity and type of fiber residue that is not digested in the small intestine and enters the colon affect the degree of fermentation (33). Psyllium, which is soluble and poorly fermentable, is fermented by gut microbes down the length of the colon to create SCFA. Resistance starch (RS) ferments more intimately in the colon because it is more fermentable and less soluble (or insoluble, depending on the type of RS) (34). Certain microorganisms in the gut specialize in the breakdown and fermentation of particular fermentable fibers (35).

Dietary fiber promotes fecal SCFAs, particularly butyrate, which was followed by improvements in glucose homeostasis (36). It is the principal source of energy for colonic epithelial cells to sustain their growth and integrity (37). Butyrate contributes to host health through having anti-inflammatory and antioxidant properties that provide benefits (38) and prevent diseases such as colorectal cancer (39, 40), diabetes, and obesity (41). Butyrate concentration mainly depends on the quantity and quality of dietary fiber reaching the colon (42). Research has shown that higher butyrate concentrations in human feces are associated with greater fiber intake (43). Foods rich in dietary fiber, such as nuts, fruit, vegetables, and cereal, are also linked to a greater abundance of SCFA producers in the human gut microbiota (44).

Optimal dietary fiber consumption, whether from foods or supplements, helps with weight loss and has positive consequences (45, 46). Most fibers reduce plasma total and low-density lipoprotein cholesterol (47). Intake of a high dietary fiber diet or wholegrain cereals lowers the risk of heart diseases (48, 49). Dietary fiber shows a significant effect as a laxative, helping reduce blood cholesterol and blood glucose levels (50). Dietary fiber is primarily used for controlling diarrhea and constipation. Different cereals and vegetables, such as cereals, gum guar, psyllium husk, and oat, are used as soluble and insoluble fibers; however, fibers from legumes, fruits, and vegetables are preferred in various metabolic syndromes (51).

Among the dietary fibers, psyllium (*Plantago ovata*) husk fiber is water soluble and derived from psyllium seed, promoting the intestinal flora. It is globally used as the best source of dietary fiber, either as functional food or supplements. Psyllium is a highly water-soluble fiber source and readily fermentable, which thereby causes less abdominal bloating (52). Psyllium husk fiber increases insulin sensitivity in a healthy individual, hinders glucose absorption, decreases postprandial glucose concentrations in the blood (53), and is an effective supplement for decreasing CVD (54) and blood pressure (55). Psyllium is a common fiber supplement widely used due to its affordability and well-tolerated than other fiber source supplements (56). It improves the blood lipid profile and acts as a bowl regulator (57). Studies suggest a strong association between psyllium husk fiber and inflammation. Increased intake of psyllium fiber further exerts an appreciable effect on risk factors for developing cancers, especially breast cancer (58, 59).

## 2 Materials and method

### 2.1 Inclusion and exclusion criteria

**Figure 1:**
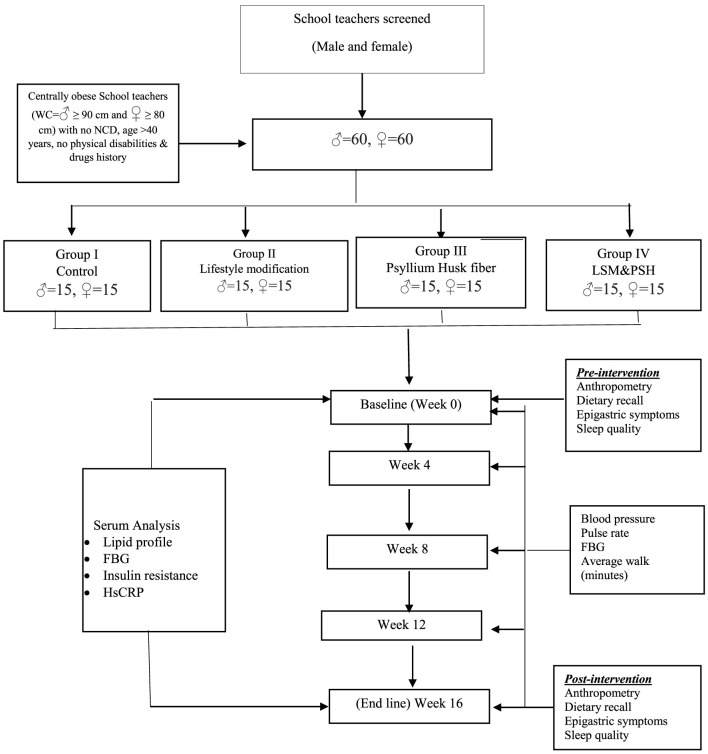
Intervention flowchart.

School teachers aged 40–60 years (men and women) with central obesity (where central obesity for Asians is defined as men having ≥ 90 cm waist circumference (WC) and for women ≥ 80 cm WC) (60–62), with no history of any chronic disease such as hypertension, diabetes, cardiovascular diseases, consumption of any regular medication, food allergies, smoking, or physical disabilities impairing the food intake and mobility, qualified the inclusion criteria. Pregnant or lactating female school teachers were also excluded during screening. Subjects with an allergy to psyllium husk fiber, a history of drug abuse, or any psychological or emotional disorder that might prevent the completion of the study were also excluded. Intervention flow chart is summarized in Figure 1.

### 2.2 Recruitment of the subject

Out of 206 screened school teachers, 185 were eligible based on waist circumference (men > 90 cm and women > 80 cm). In terms of predicting cardiovascular and metabolic risk, WC and BMI have a substantial correlation. BMI is simple to calculate, but it does not differentiate between lean and fat masses (63). Among the 185 eligible school teachers, 33 were excluded from the study. In total, 22 school teachers (eight men and 13 women) refused a blood sample (due to syringe needle allergy/phobia), and 11 (six men and five women) were not willing to use psyllium husk regularly due to some myths and personal reasons. In total, 76 men and 76 women school teachers (considering that 10% dropped out for many reasons) were enrolled in the study.

### 2.3 Study design

A group of 120 school teachers (60 men and 60 women) was divided into four subgroups for 16 weeks of an interventional study. One group was kept as control, while the other three were assigned interventions. One group was assigned the intervention of lifestyle modification (LSM), another group was assigned the intervention of 5 g of psyllium husk fiber (PSH) twice daily, and the third group was assigned the combination of LSM and PSH. Each group consisted of 30 subjects, equally divided by gender (15 men and 15 women). Maximum homogeneity in terms of the geographical, social, and financial conditions among and within groups was maintained (summarized in Table 1).

**Table 1:** Study groups assignment

**Group (*n* = 120)**	**Role**	**Intervention**	**Compliance**
Group I (*n* = 30)	Control	No intervention	NA
Group II (*n* = 30)	Lifestyle modification (LSM)	(Diet, physical activity, and behavior modification). 1. Minimum 30 min walks 2. Inclusion of fruits and vegetables (mini 5 servings/day) 3. Low salt and refined sugar intake	Appendix III a Appendix III b Appendix IV
Group III (*n* = 30)	Psyllium husk fiber (PSH)	30 min before breakfast and dinner, consume 5 g of psyllium husk fiber (in swollen form) twice daily.	Demonstration of the preparation of psyllium husk fiber. Reminder texts and calls. Collection of empty zip lock PSH bags.
Group IV (*n* = 30)	The combined group of LSM&PSH	a. Diet, physical activity, and behavior modification 1. Minimum 30 min walks 2. Inclusion of fruits and vegetables (mini 5 servings/day) 3. Low salt and refined sugar intake b. 30 min before breakfast and dinner, consume 5 g of psyllium husk fiber (in swollen form) twice daily.	Appendix III a Appendix III b Appendix IV Demonstration of the preparation of psyllium husk fiber. Reminder texts and calls. Collection of empty zip lock PSH bags.

### 2.4 Intervention

An informed consent form was signed by each subject, explaining the data privacy and the subject's obligations. School teachers were actively involved in a group discussion about their current food behavior based on locally available food. They were probed about the importance of situation-specific nutrients and the development of a particular diet. A guided tool was developed from the Pakistan Dietary Guidelines for Better Nutrition 2018, covering the nutritional recommendations for subjects (64).

A written and informed consent explaining the study's importance and the subject's obligation was signed under the approval of the ethical committee from the enrolled school teachers.

### 2.5 Abdominal and epigastric health symptoms

A Dutch-developed English version of the questionnaire (65) assessed abdomen and epigastric health. The questionnaire depicts the health symptoms of the gastrointestinal tract throughout the last 4 weeks, rated from 0 to 6, where 0 represents no complaints and six refers to the severity of symptoms. In a physical demonstration, the questionnaire explained the severity of GI symptoms on a scale of 0–6. The subject was asked about their symptoms and probed for their severity on a defined scale of 0–6. Pre- and post-intervention questionnaires about GI symptoms were filled out during face-to-face seating, and the subjects shared their past 1-month experiences. The subjects' symptoms were assessed using the wide, general phrase stomach discomfort. GI health is discomfort or pain in the lower or upper abdomen, depending on abdominal and epigastric sensations. Epigastric pain was classified as an intense burning or gnawing pain in the mid-epigastric region, frequently associated with other upper GI symptoms and perhaps caused by back radiation. Pain in the retrosternal region (heartburn) is caused by stomach acid, which frequently progresses to the neck and worsens with bending over, that is frequently associated with eating fatty foods, chocolate, or even restrictive clothes. Regurgitation, or a spontaneous reflux of stomach acid or contents into the esophagus and occasionally into the mouth, can grow into a pitiful condition, particularly in the morning or after consuming a large amount of oily meal. Belching was once thought to be the audible escape of air from the stomach via the mouth. Additionally, bloating, or abdominal distension, was defined as a condition in which the belly feels full and constricted, not just after eating, and was frequently interpreted as an abnormal amount of intestinal gas (66).

### 2.6 Sleep quality analysis

The Pittsburgh Sleep Quality Index (PSQI) questionnaire was used to assess the preceding month's sleep quality of school teachers. The Pittsburgh Sleep Quality Index (PSQI) is a self-report questionnaire that evaluates sleep quality and disruptions over 1 month. There are seven “component” scores generated by 19 individual items: subjective sleep quality, sleep latency, sleep duration, habitual sleep efficiency, sleep disruptions, usage of sleeping medication, and daytime dysfunction. The sum of the scores for these seven components results in a single global score (67). All questions were based on a subjective sleep quality rating scale ranging from 0 to 3 (a score of 0 indicates that it has not happened in the last month, 1 indicates that it has happened less than once a week, 2 indicates that it has happened once or twice a week, and 3 indicates that it has happened three or more times a week). The component scores are added together to generate a global score (range 0–21). Higher scores suggest poorer sleep quality. Subjects were asked about their sleep quality, sleep duration, sleep efficiency, sleep disruption, sleep medicine, and sleep-related daily dysfunction using the PSQI questionnaire scale at both the beginning and end of the study.

### 2.7 Preparation and consumption of psyllium husk fiber

The finest quality psyllium husk fiber from Sinhala Herbs (23, Industrial Estate, Neemuch, Madhya Pradesh, India) was procured. The subjects were instructed regarding proper preparation and consumption. Based on previous studies (68, 69), 5 g of psyllium husk fiber in a zip-lock bag was provided to the subjects in the PSH and LSM&PSH groups. In a practical demonstration, the subjects were briefed about preparation and consumption. Psyllium husk fiber was immersed in half a glass of warm water/milk and waited for 10–15 min. The psyllium husk fiber absorbed sufficient water and swelled to the maximum to form a gel. The subject consumed the swollen gel psyllium husk fiber with one glass of warm water to clear the epigastric tract of any fiber debris and to avoid choking. The subjects were advised to consume 5 g of psyllium husk fiber twice daily, 30 min before breakfast and 30 min before dinner.

### 2.8 Lifestyle modification

#### 2.8.1 Physical activity

The subjects in the lifestyle modification and combined lifestyle modification groups, along with psyllium husk fiber, integrated walking into their daily lives. Based on the WHO guidelines, a minimum walk of 150–300 min of moderate-intensity aerobic physical activity or 75–150 min of vigorous-intensity aerobic physical activity throughout the week (70) was suggested. The subject's average weekly data was recorded on the diary page (Appendix A).

#### 2.8.2 Dietary modification

A 24-h dietary recall for any 3 random days was collected with the support of portion size estimation using standard household measures like the cup, bowl, and spoon (data reported somewhere else). The five-step multiple-pass method was used for a 24-h dietary recall, including the quick list of food consumed on any random day, the forgotten food list, the time and occasion of food consumed, the cooking method (fried, boiled, roasted, and steamed), and the amount of food consumed. The subjects were interviewed in a relaxed, conducive environment about their meal intake and portion size and probed for food taken in complex forms. Portion sizes consumed were entered in gram weights, and the nutrient composition of the food consumed was calculated using NutriSurvey (Nutrisurvey for Windows. Copyright 2007. Dr. Juergen Erhardt, SEAMEO-TROPMED RCCN, Indonesia) (71). Total calories, protein, carbohydrates, fats, dietary fiber, vitamins A, E, B1, B2, B6, folic acid, vitamin C, sodium, potassium, calcium, magnesium, phosphorus, iron, zinc, polyunsaturated fatty acid (PUFA), and cholesterol were calculated.

#### 2.8.3 Behavior change toward healthy and nutritious food

The 5A tool (Appendix B) was used to assess the dietary behavior of the subjects and align with the dietary guidelines of Pakistan (Appendix C) for food exchange choices and compliance. For subject awareness, a standard format (Appendix D) of dietary messages adopted from the dietary guidelines of Pakistan was developed and shared in groups. Subjects were oriented in one-to-one and group discussions and during follow-up visits.

### 2.9 Intervention compliance

Weekly and monthly follow-up visits were planned to their respective schools for efficient compliance with the intervention protocols. Each teacher's progress was noted, and bottlenecks were sorted out for compliance. An average walk-in minutes of 4 weeks was asked and reconfirmed with the Android or iOS health software record available on certain school teachers' smartphones. The count of empty sachets confirmed regular consumption of psyllium husk fiber at the end of each week/month. All the subjects were in close liaison via text/WhatsApp (group messages). The reminder messages were sent to the subjects in intervention groups before breakfast (06:00 a.m.) and dinner (05:00–06:00 p.m.).

### 2.10 Statistical analysis

For the baseline, an ANOVA was used to compare the means of the four groups. Student's *t*-test was used to determine the mean difference between pre- and post-interventions. The post-intervention effect was analyzed using ANCOVA after adjustment for age, gender, and baseline.

### 2.11 Ethical approval

Ethical committee approval was sorted out for the school teachers who participated in this interventional study voluntarily under the ethical committee approval HN-HREC-2020-0012, dated 26 August 2020, and signed the consent form.

## 3 Results

### 3.1 Pre-existing abdominal and epigastric health conditions

**Figure 2:**
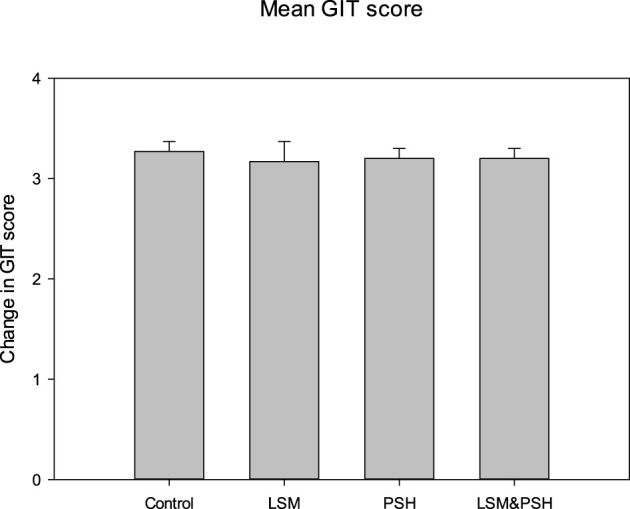
GIT mean symptom score of the study subjects at baseline. A one-way analysis of variance was employed for the study of differences between variables (*P* = 0.05). PSH, psyllium husk fiber; LSM, lifestyle modification; GIT, gastrointestinal tract.

Figure 2 explains the abdominal and epigastric health symptoms on a widely used and validated questionnaire to evaluate GI symptoms. Gastrointestinal health (GIT) was evaluated on a Likert scale (0–6), where 0 represents no symptoms and 6 reflects unbearable conditions (65). Group-wise mean GIT score variance was non-significant (*P* = 0.985). The mean GI score of symptoms perceived in the control group was 3.27 ± 0.5, the LSM mean GIT score was 3.17 ± 1.6, the PSH mean score was 3.2 ± 0.9, and the LSM&PSH group had a mean GIT score of 3.2 ± 0.7 at baseline. The mean GIT score of symptoms was non-significant (*P* = 0.86) between men and women; the mean GI scores of men and women were 3.2 ± 0.9 and 3.23 ± 1.07 at baseline, respectively (Table A1).

### 3.2 Sleep quality assessment at the baseline

**Figure 3:**
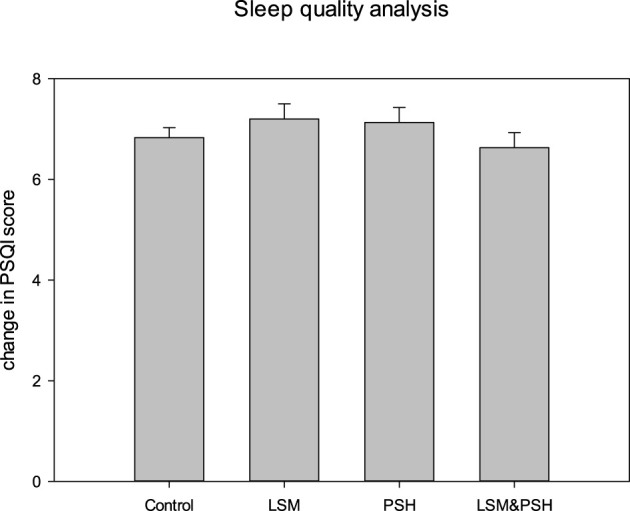
Sleep quality analysis based on the mean PSQI score. A one-way ANOVA was performed for the comparisons (*P* = 0.05). PSQI, Pittsburgh Sleep Quality Index; PSH, psyllium husk fiber; LSM, lifestyle modification.

Figure 3 shows the Pittsburgh Sleep Quality Index (PQSI), an established questionnaire for sleep quality analysis. The PSQI is composed of 19 items that produce a global score for sleep quality, and the study participants were evaluated at baseline on the following seven components: sleep quality, sleep latency, sleep length, sleep efficiency during habitual sleep, sleep disturbance, usage of sleeping medicine, and daytime dysfunction (72).

As measured by the PSQI score, the quality of sleep did not differ significantly (*P* = 0.661). The LSM group had the highest PSQI score at baseline, while the LSM&PSH group had the lowest.

A significant difference (*P* < 0.05) was observed between men and women sleep quality at baseline. Men have poor sleep quality, with a mean PSQI score of 7.2 ± 1.05, compared to women, with a mean PSQI score of 6.6 ± 1.32 (Table A2).

### 3.3 Post-intervention mean GIT health status-group-wise trend

**Figure 4:**
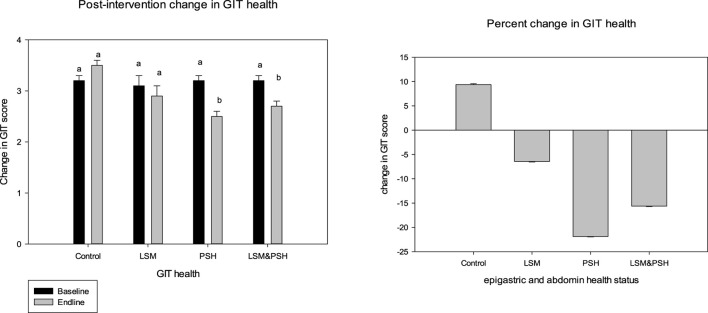
Post-intervention GIT health status. A paired sample *t*-test was employed to evaluate the difference between the pre- and post-intervention (*P* = 0.05). GIT, gastrointestinal tract; LSM, lifestyle modification; PSH, psyllium husk fiber. Result between similar alphabets remains non significant.

Figure 4 shows the gastrointestinal tract (GIT) health after the intervention in the control and intervention groups. Both psyllium husk fiber alone and in combination with lifestyle changes had a substantial influence on GIT health. The psyllium husk fiber group showed the maximum effect (*P* < 0.05) with a mean of −0.7 ± 0.1 (−22%), and the mean GIT score of the LSM&PSH group was −0.5 ± 0.1 (−16%). In intervention groups, the LSM group had a minimal effect (−6%) compared to the PSH and LSM&PSH groups. However, the GIT score increased by 9% in the control group.

A significant effect has been observed in both genders of GIT health. Women showed the highest response in relieving GIT scores compared to men. The mean GIT score of women was −0.3 ± 0.1 (−9%), and the post-intervention mean GIT score of men was −0.2 ± 0.1 (−7%) (Table A3).

### 3.4 Mean adjusted changes in epigastric and abdominal health

Table 2 explains the post-intervention effect of treatment on GIT health. After adjusting for age, gender, and baseline, a significant effect was observed in the intervention groups. The PSH group showed maximum relief (−22%) in GIT symptoms. Similarly, the LSM&PSH group showed a −16% improvement and the LSM group showed a 6% improvement in GIT symptoms.

**Table 2:** Post-intervention means GIT health status.

**Change in variables**	**Adjusted mean** ±**SEM (95% CI) (*****n*** = **30**				***P*-value**
	**Control**	**LSM**	**PSH**	**LSM&PSH**	
GIT	3.50 ± 0.14 (3.79; 3.21)a	2.99 ± 0.14 (3.27; 2.70)ab	2.49 ± 0.14 (2.78; 2.20)b	2.71 ± 0.14 (3.0; 2.42)ab	< 0.001

The following values are assigned to covariates appearing in the model: age = 45.91, gender = 1.50, GIT baseline score = 3.2167 (P < 0.05). GIT, gastrointestinal tract; PSH, psyllium husk fiber; LSM, lifestyle modification. R-squared = 0.437 (adjusted R-squared = 0.407). Design: intercept + age + gender + GIT baseline score + study group. Similar alphabet in the row shows non significant relation.

### 3.5 Post-intervention sleep quality analysis: the group-wise trend

**Figure 5:**
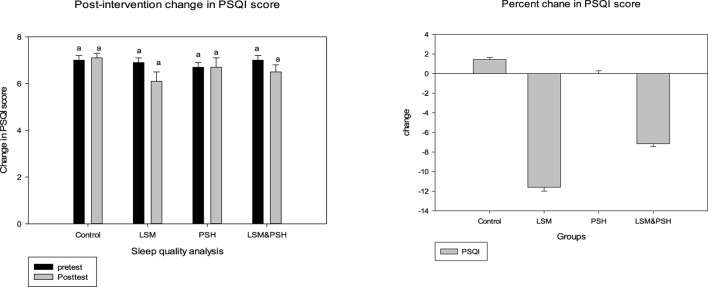
Post-intervention trend based on the mean PSQI score. A paired sample *t*-test was employed to evaluate the difference between the pre- and post-intervention (*P* = 0.05). PSQI, Pittsburgh Sleep Quality Index; LSM, lifestyle modification; PSH, psyllium husk fiber. Similar alphabet represent the non significant relation among the group.

Figure 5 shows the effect of the intervention on sleep quality based on the Pittsburgh Sleep Quality Index (PSQI) score, which ranges from 0 to 21. A non-significant change was observed in the sleep quality of the subjects; however, an improvement was observed in the LSM group compared to the other groups, with a mean difference in the PSQI score of −0.7 ± 0.4 (−12%). The PSQI score was reduced by 7% in the LSM&PSH group, while the PSH group's PSQI score remained unchanged. Gender-wise sleep quality analysis showed a non-significant change in the sleep quality of men (4%) but a significant change in that of women, which thereby demonstrated a decrease of 15% PSQI score (Table A4).

### 3.6 Effect of intervention on sleep quality (mean PSQI score)

Table 3 explains the effect of the intervention on sleep quality between the groups based on the PSQI score. The intervention's effect, when compared to the control group and after controlling for age, gender, and baseline, was not statistically significant (*P* = 0.205). PSQI scores increased in the control group and remained the same in the PSH group, while they decreased in the LSM and LSM&PSH groups. The maximum effect was observed in the LSM group (−12%), followed by the LSM&PSH group (−7%).

**Table 3:** Adjusted mean PSQI score.

**Change in variables**	**Adjusted mean** ±**SEM (95% CI) (*****n*** = **30)**				***p*-value**
	**Control**	**LSM**	**PSH**	**LSM&PSH**	
PSQI	7.04 ± 0.32 (7.68; 6.41)a	6.16 ± 0.32 (6.8; 5.53)b	6.84 ± 0.32 (7.48; 6.20)ab	6.40 ± 0.32 (7.05; 5.76)ab	0.205

The following values are assigned to covariates appearing in the model: age = 45.91, gender = 1.50, GIT baseline score = 6.9333 (*P* < 0.05). GIT, gastrointestinal tract; LSM, lifestyle modification; PSH, psyllium husk fiber. *R*-squared = 0.342 (adjusted *R*-squared = 0.307), design: intercept + gender + age + PSQI baseline score + study group. Similar alphabet in the row shows non significant relation.

## 4 Discussion

Post-intervention findings showed that consumption of 10 g of psyllium husk fiber twice a day, 30 min before breakfast, and dinner in soaked form, improved the GIT symptoms, with the most significant improvement observed in the PSH group, followed by the combined group of LSM&PSH. However, gender-wise data showed that maximum improvement was observed in the female group compared to the male group of the study. Based on the PSQI score, the study revealed that the effects of either psyllium husk fiber alone or combined with lifestyle modification were non-significant. However, an improvement has been observed in the LSM group.

Jalanka et al. (73) assessed the role of psyllium husk fiber in the wellbeing of GIT health by examining the role of psyllium husk fiber on the fecal microbiota, which plays a crucial role in gut physiology. In a short study, he shared that psyllium has a small but significant effect on the microbial composition of healthy adults while having a more significant effect on the microbial composition of constipated subjects.

Dietary fiber plays a significant role in lowering the risk of colorectal cancer. The mechanism involved the dilution of fecal carcinogens, quicker gut transit time, bonding of carcinogenic bile acids, and alteration in the microbiota composition and microbial metabolites such as short-chain fatty acid production (74). Bovenschen et al. (65) reported the same GIT symptoms based on this questionnaire. They reported that the severity score of GI symptoms (type and severity) at the end line compared with the baseline and difference (post-base) showed the trend in GIT health (severity or improvement).

Marlett et al. (75) studied the psyllium husk fiber (15 g/day) effect on a stool alone. They reported that psyllium considerably improved the apparent viscosity, stool wetness, and wet and dry stool weight of an aqueous stool extract. Compared to other study subjects who consumed other dietary fiber and control, the subjects with psyllium husk fiber showed a significant improvement in gut health and open defecation.

Similarly, Marteau et al. (76) reported that the improved digestibility of psyllium husk fiber and its fecal bulking effect improve gut transit time and gas excretion. The positively impact-producing short-chain fatty acid concentration in stool provides the best medium for intestinal flora growth. Desai et al. (77) studied the actual benefits of dietary fiber in the context of irritable bowel syndrome. They related its influence to the microbiota and the maintenance of mucosal integrity.

PSQI is widely used to assess an individual's sleep quality without medication. Subjects with a score of more than five are considered to have poor sleep quality, while less than five PSQI scores reflect good quality sleep (67). Katagiri et al. (78) evaluated sleep quality using the PSQI questionnaire. According to their findings, poor sleep quality is substantially associated with consuming more sweets and beverages and fewer fruits and vegetables. Epidemiological studies suggest a bidirectional relationship between sleep and overall dietary patterns. Most notably, dietary fiber, whole grains, fruits, and vegetables are associated with longer sleep duration, better sleep quality, and fewer insomnia episodes (79).

Sleep disturbances have been linked to hypertension, stroke, and obesity via increased ghrelin and decreased leptin levels, impaired glucose tolerance, anxiety and depression, increased evening cortisol production, and higher inflammatory markers (80, 81). Inadequate sleep delays the circadian melatonin phase while also causing the circadian waking time phase to begin sooner. Sex differences revealed that women, not men, maintained weight during adequate sleep, whereas poor sleep impaired dietary control and caused weight gain in women. Liang et al. (82) studied the dietary approaches to stop hypertension (DASH) and their association with sleep quality. An inverse relationship was found between the DASH score and poor sleep-related daytime dysfunction. The fiber DASH component was most notably associated with better sleep quality and inversely related to sleep-related daytime dysfunction.

Grandner et al. (83) assessed the national data from the US adult survey to quantitatively demonstrate the relation between dietary fiber intake and sleep quality. Subjects with decreased intake (13.2 ± 10.1 g) have less sleep duration of < 5 h, while subjects who consumed 14.2 ± 8.7 g and 15.9 ± 10.9 g have 5–6 h and 9+ h of sleep, respectively. High-fiber foods like fruits, vegetables, and cereals encourage the production of short-chain fatty acids (SCFA) in the human gut microbiota (44). These gut micro-biotas enhance sleep (84) and modulate the host circadian clock (85), which in turn maintains mammalian homeostasis and rhythmic physiology such as sleep-wake cycle, eating, and fasting (86). Contraction with the literature augments the need for further studies to correlate the other factors influencing sleep quality and suggest modifications in the PSQI questionnaire in light of modern technological interference in sleep quality components.

The association of specific diets such as high energy intake or different food nutrient intakes at different levels of carbohydrates, fats, and protein on sleep quality and duration is unclear. Previous studies have shown that increased consumption of energy intake induces insufficient sleep and could induce body weight gain (87, 88), while others reported that less energy intake had a profound effect on insomnia (89).

## 5 Summary and conclusion

A 16-week interventional study was conducted to assess the effect of lifestyle modification with and without psyllium husk fiber on the abdomen, epigastric symptoms, and sleep quality. In total, 120 school teachers with equal gender bifurcation were divided into four subgroups (*n* = 30), a control group, and an intervention group. The enrolled subjects were oriented about the intervention execution and compliance, encouraged to participate in one-to-one and group discussions, and signed informed consent.

In multiple seating, GIT health status, and sleep quality analysis were recorded using validated questionnaires. GIT health symptoms improved post-intervention in the PSH and combined LSM&PSH groups. GIT health symptoms improved by 22% in the PSH group and 16% in the LSM&PSH group; however, a non-significant 6% improvement was noted in the LSM group. Gender-wise data showed a significant improvement in GIT health in both genders. However, women improved more than men (9 vs. 7%). Based on the PSQI score, the intervention has a non-significant effect on sleep quality, yet the LSM group showed the highest effect on sleep quality compared to the PSH and the LSM&PSH groups. Gender-wise PSQI analysis showed a non-significant effect of the intervention on the sleep quality of men but a highly significant effect on the sleep quality of women.

The study concluded that psyllium husk fiber significantly affects the abdomen and epigastric health. At the same time, lifestyle modification is more potent in enhancing the subjects' sleep quality. Further studies are suggested to include the technological effect on sleep quality by modifying the PSQI questionnaire.

## 6 Limitations

Adherence to the protocol intervention and myths about psyllium husk fiber were challenging at inception. Dietary counseling, sparing time for a dedicated walk, and reporting by subjects in intervention groups on a regular basis pose a challenge for acceptance. Individual follow-up, protocol compliance, and reporting require additional effort along with the intervention. Dietary behavior modification for the subject and in support of the family increase the budget of households. Lack of technological awareness and usage for measuring physical activity added to the extra burden of proper reporting on daily diaries, which required in-depth training and continuous follow-up to minimize bias.

## 7 Strength

This is a detailed study in the region, particularly in the teacher community. Through individual and group counseling, awareness about the lifestyle modification and the role of psyllium husk fiber, apart from anti-constipation, develops the social desirability and willingness of the subjects. Including school teachers as an influential segment increased the generalizability of the study. Regular follow-up and subject's capacity building on reporting formats and adherence to the lifestyle modification and consumption of psyllium husk fiber protocol improve the study quality, increase the subjects' interests in learning and outcomes, reshape the work-life balance, develop dietary modification behaviors, and promote more health and nutrition concerns.

## 8 Recommendations

To sustain the intervention achievements, adherence to lifestyle modification and establishing a strong work-life balance are required. Further studies are suggested to include < 40-year-old adults, pre-diabetic, and hypertensive subjects from the general population. The health sector and practitioners are involved in disseminating the right dietary approaches and compliance. Mass-level nutrition education is a preventive tool for non-communicable diseases. The inclusion of objective sleep assessment methods, such as actigraphy, has to be included in future studies for a comprehensive evaluation of sleep quality.

## Data availability statement

The raw data supporting the conclusions of this article will be made available by the authors, without undue reservation.

## Ethics statement

The studies involving humans were approved by Ethical Committee of the Department of Human Nutrition, University of Agriculture Peshawar HN-HREC-2020-0012. The studies were conducted in accordance with the local legislation and institutional requirements. The participants provided their written informed consent to participate in this study. Written informed consent was obtained from the individual(s) for the publication of any potentially identifiable images or data included in this article.

## Author contributions

AB: Investigation, Methodology, Writing – original draft, Writing – review & editing. MS: Formal analysis, Data curation, Writing – review & editing. FA: Funding acquisition, Formal analysis, Writing – review & editing. EI: Data curation, Visualization, Writing – review & editing. HA: Software, Validation, Writing – review & editing.
