# Erratum: Role of dietary fiber and lifestyle modification in gut health and sleep quality

**DOI:** 10.3389/fnut.2024.1429023

**Published:** 2024-05-14

**Authors:** Frontiers Production Office

**Affiliations:** Frontiers Media SA, Lausanne, Switzerland

**Keywords:** sleep analysis, gastrointestinal tract, PSQI, GIT score, psyllium husk fiber, lifestyle modification, dietary fiber

Due to a production error, there was a mistake in the affiliations for “Hijaz Ahmad”. In addition to affiliation(s) 4 and 5, they should also have “Section of Mathematics, International Telematic University Uninettuno, Rome, Italy and Near East University, Operational Research Center in Healthcare, Nicosia, Türkiye”.

The publisher apologizes for this mistake. The original article has been updated.

