# Corrigendum: Role of dietary fiber and lifestyle modification in gut health and sleep quality

**DOI:** 10.3389/fnut.2024.1429445

**Published:** 2024-09-05

**Authors:** Amjad Ali Bacha, Muhammad Suhail, Fuad A. Awwad, Emad A. A. Ismail, Hijaz Ahmad

**Affiliations:** ^1^Department of Human Nutrition, The University of Agriculture Peshawar, Peshawar, Pakistan; ^2^Amir Muhammad Khan Campus Mardan, The University of Agriculture Peshawar, Peshawar, Pakistan; ^3^Department of Quantitative Analysis, College of Business Administration, King Saud University, Riyadh, Saudi Arabia; ^4^Center for Applied Mathematics and Bioinformatics, Gulf University for Science and Technology, Mishref, Kuwait; ^5^Department of Computer Science and Mathematics, Lebanese American University, Beirut, Lebanon; ^6^Section of Mathematics, International Telematic University Uninettuno, Rome, Italy; ^7^Near East University, Operational Research Center in Healthcare, Nicosia, Türkiye

**Keywords:** sleep analysis, gastrointestinal tract, PSQI, GIT score, psyllium husk fiber, lifestyle modification, dietary fiber

In the published article, the Acknowledgment section was omitted. The Acknowledgment section appears below.

