# Supplementary material for: Role of dietary fiber and lifestyle modification in gut health and sleep quality
Source: Front Nutr. 2024 Apr 3;11:1324793. doi: 10.3389/fnut.2024.1324793 (PMC11022964; doi:10.3389/fnut.2024.1324793)
Supplement: Supplementary file 1 [file Table_1.pdf]

APPENDIX

Table 1. Gender-wise mean GIT symptoms at baseline

Gender wise	Mean±SD		P Value
	Male (n=60)	Female(n=60)	
**GIT score**	**3.2±0.9**	**3.23±1.07**	**0.86**

A one-way ANOVA was employed to compare the variables (P0.05).

Table 2. Gender-wise mean PSQI score at baseline

Gender wise	Mean±SD		P Value
	Male (n=60)	Female(n=60)	
PSQI score	7.2±1.05	6.6±1.32	<0.05

A one-way ANOVA was employed to compare the variables (P0.05), PSQI; Pittsburgh sleep quality index.

Table 3. Gender-wise gastrointestinal tract health (GIT) status

Profile	Gender	Mean±(SEM)				P-value
		N	Pretest	Posttest	Change (95% CI)	
GIT	Male	60	3.2±0.1	2.9±0.9	-0.3±0.1(-0.5; -0.05)	<0.05
	Female	60	3.2±0.1	2.9±0.1	-0.3±0.1(-0.5; -0.02)	<0.05

A paired sample T-test was employed to evaluate the difference between the pre- and post-test (P0.05)

GIT; gastrointestinal tract,

Table 4. Gender-wise, the Pittsburgh sleep quality index (PSQI) mean difference status

Profile	Gender	Mean±(SEM)				P-value
		N	Pretest	Posttest	Change (95% CI)	
PSQI	Male	60	7.2±0.1	7.5±0.2	0.33±0.2(-0.1; 0.8)	0.163
	Female	60	6.6±0.1	5.6±0.2	-0.9±0.2(-1.4; -0.4)	<0.001)

A paired sample T-test was employed to evaluate the difference between the pre- and post-test (P0.05)

PSQI; Pittsburgh sleep quality index,

Appendix-A

Daily physical activity

Name_________________ ID__________________ Date______________

1. Have you done any physical activity last week? If yes: Daily walk/jogging

Day	Walk/jogging	Duration (time)	Distance (Km)	Remarks
Day 1				
Day 2				
Day 3				
Day 4				
Day 5				
Day 6				
Day 7				

a. Have you worked in garden? Yes ☐ No ☐

   If yes:

   i. How long? ______________________

   ii. How much you were physically active? ______________________

b. Did you go for shopping? Yes ☐ No ☐

   If yes:

   i. How long(minutes) ___________

c. Did you offer prayer in Masjid? Yes ☐ No ☐

   If yes:

   i. Total distance for one time prayer (home-Masjid-home) you cover

      i. Km__________Time (minutes) ____________

d. Any other physical activity?

   If yes:

   I. How long(minutes)_____________________

   II. How physically active you been?

   Less active ☐ Moderate active ☐ more active ☐

e. Have you done housekeeping?

   If yes:

   I. How long(minutes)_____________How physically active have you been? ______________

Appendix-B

Behaviour change

5As	What to say/do and how to say/do it (for example)
Ask	How many portions of fruit and vegetables do you eat each day?
Advice	Eat at least 5 portions of fruit and vegetables per day.<br><br>Eat a variety of fruits, vegetables, legumes (lentils, beans), nuts and whole grains (unprocessed maize, millet, oats, wheat, and brown rice), starchy tubers or roots (potato, yam, taro or cassava) and foods from animal sources (meat, fish, eggs and milk).
Assess	Assess the individual willingness towards acceptance and probed for the worries?
Assist	Help them to set goals and make a plan to start introducing some changes to their eating habits. The most apparent and easy goal was the weight and waist circumference.
Arrange	Follow up by different method, like visit to school, by the way meetings, contact through text messages and voice calls, sharing of information via whatsapp and involving them in active discussion.

Toolkit for delivering the 5A's and 5R's brief tobacco interventions in primary care. Geneva: World Health Organization; 2014 (http://www.who.int/tobacco/publications/ smoking_cessation/9789241506953/en/, accessed 31 March 2017).

Appendix-C

DIETARY MESSAGES

Dietary messages have been developed in the light of scientific knowledge and epidemiological evidence to address nutritional and public health problems and to reduce and control the incidence of diseases and premature deaths. Efforts are made to make the dietary messages simple, easy, specific, comprehendible, meaningful, sensitive and culturally appropriate.

Maintain normal body weight by consuming all food groups and performing regular physical activity

Maintaining a normal (healthy) bodyweight through healthy dietary practices and regular exercise is vital for longevity and wellbeing, as both underweight and overweight are associated with increased prevalence of morbidity and mortality. Dietary diversification with regular exercise can minimize the adverse effects of obesity and overweight on health. Individuals who maintain normal body weight and are physically active have the lowest incidence of chronic disease and mortality.

Half of your daily cereals intake should include whole grains

Whole grain cereals and pulses are good sources of providing energy, dietary fibre, proteins, carbohydrates, vitamins, minerals and antioxidants to the human body. They have been associated with reducing the risk of developing hypertension, metabolic syndrome, coronary heart disease, diabetes, cancers and other diseases. Consumption of refined wheat and wheat products (pasta, noodles, white bread and other bakery products), rice and other cereals with low or negligible levels of dietary fiber have been linked to increased risk of digestive problems and colorectal cancers. About 45-65% of total calories shall come from 92 carbohydrate containing foods.

Eat five servings of fresh vegetables and fruits a day

Leafy green, deep yellow and red vegetables and fruits are rich sources of vitamin A, C, folic acid, iron, dietary fiber, potassium and antioxidants. They are free of fat and cholesterol, low in calories and contain many other beneficial compounds that prevent from overweight, obesity and incidence of chronic diseases and cancers. Five servings of vegetables and fruits per day are recommended for optimum health.

Take two to three servings of milk and milk products in a day

Milk and milk products are rich sources of calcium, phosphorous, magnesium, vitamin D, lactose, protein and fat, which are essential for skeletal growth and development. Adequate consumption of dairy products has been associated with strong bones and increased cognitive and physical outputs. For individuals who are overweight and obese or having cardiovascular diseases, skim milk is a preferred choice. Inadequate consumption of dairy products and lack of exposure to sunlight leads to rickets, osteomalacia and osteoporosis later in life. Two to three servings of milk and milk products per day are recommended to meet calcium and other essential nutrient requirements of the body.

Consume meat and meat products, fish and eggs in moderation

Meat and meat products, fish and eggs are rich in proteins, iron, zinc, vitamin A, vitamin B12 and integral component of hormones, enzymes and body immune system. Meat without fat (lean meat) is recommended over fatty meat (non-lean meat). Fish is recommended at least once in a week to supply protein and essential fatty acids to the body for growth and brain development. Excess amount of beef, sheep and goat meats shall be avoided to prevent high intake of saturated fat and cholesterol. Consumption of non-lean meat has been associated with increased cardiovascular diseases, breast and prostate cancers. Two to three servings of meat and meat products, chicken, fish and eggs per day are recommended to fulfill protein and other essential nutrient requirements of the body.

Encourage consumption of pulses to attain healthy growth

Pulses are rich in protein, dietary fibre, vitamins, minerals and essential amino acids particularly lysine, which complement the nutrients profile of wheat, rice and other cereals when consumed together. The protein content of pulses are almost equivalent to meat protein and therefore, could be used as a substitute where there is a shortage of meat or expensive to afford. They could be used as a preventive and therapeutic diets, as they tend to lower the risk of chronic non-communicable diseases i.e., hyperlipidemia, heart diseases, hypertension, diabetes and cancers.

Consume fortified flour, grains and their products

Fortified wheat flour and cereals are good sources of energy, proteins, vitamins and minerals. Use of fortified foods should be preferred over non-fortified foods to complement nutrients intake and combat macro and micronutrients deficiencies, improve health status and reduce risk of diseases. Micronutrient fortification has led to significant improvements in micronutrient status of women and children and reduction in prevalence of goiter, neural tube defects and other associated defects.

Limit consumption of edible oil and fat in cooking

Non-communicable diseases including diabetes, hypertension, cardiovascular diseases and cancers are reaching an epidemic proportion in Pakistan. These are responsible for the increasing burden on the health care system, human sufferings and premature deaths adversely affecting productivity and socio-economic development of the country. Consumption of saturated fat and trans-fatty acids increases low density lipoproteins (LDL) and cholesterol (bad fat) that increase the risk of hypertension and cardiovascular diseases. Evidence suggests that replacement of saturated fat and trans-fatty acids, with unsaturated cooking oil reduces bad fat (LDL & cholesterol) and increases good fat (high density lipoprotein) that decreases the risk of CVDs.

Reduce sugar intake, and limit intake of soft drinks, confectionaries, bakery products and commercial fruit drinks

Sugar sweetened drinks and foods with high sugar should be limited. Soft drinks, confectionaries, bakery products and commercial fruit drinks are energy dense foods and mostly provide empty calories increasing 54 Pakistan Dietary Guidelines for Better Nutrition the risk of overweight and obesity. These foods also contain large amount of sugar. Consumption of soft drinks and commercial sweet carbonated beverages has been strongly associated with increased weight gain, diabetes and pancreatic cancer. Consumption of complex carbohydrates is preferred over simple sugars. Reduction in sugar intake, soft drinks, confectionaries, bakery products, sweets and commercial sweetened fruit drinks is recommended.

Limit salt in cooking and always use iodized salt

In Pakistan, salt is more frequently used in diets, pickles and processed foods, and a potential risk factor for hypertension and other cardiovascular diseases. A positive association has been found between salt intake, hypertension, strokes and premature deaths. Salty foods are high in sodium. Sodium is one of the main dietary contributors to hypertension (high blood pressure). Even a small (1 g per person per day) reduction in salt intake will reduce deaths from strokes and heart attacks by more than 7% in each country. Avoid sprinkling of salt on fruits (oranges, lemons, grape fruits, guava, peaches, plums etc.) and beverages (soft drinks and orange, grape, peach, plum juices). Reduction in salt intake (< 5 g/day) is recommended for optimum health.

Limit consumption of fatty foods and highly processed foods

To improve health and nutritional status, substitution of junk (empty calories) and deep fried foods with healthy foods is essential. It is well established that consumption of junk and deep fried foods promote overweight and obesity that predispose children and adults to increased occurrence of diseases, resulting in impaired productivity and economic outputs. Highly processed foods, especially packaged, ready-made foods, are often high in fat, sugar and or salt and should be avoided. Instead eating raw vegetables and fruits and choosing fresh foods instead of packaged foods will contribute to maintain healthy diet.

Change sedentary lifestyle to physically active lifestyle

Physical activity is essential for skeletal growth and development, weight management, prevention of non-communicable diseases, increased productivity and better quality of life. At least half hour or more moderate exercise (e.g. walking) per day is recommended for physical fitness/health.

Exclusively breastfeed the baby in the first six months and continue breastfeeding along with complementary feeding at least for two years

Breast milk is a complete food for the first six months of life. It provides all the essential nutrients required by the baby for growth and development and therefore, no other liquid or food is required till the age of six months. After the age of six months, breastfeeding shall be complemented by nutrient dense and better varieties of safely prepared foods according to the infant and child's age. In order to allow child skin to form vitamin D, children over the age of six months should be exposed to sunlight in the early morning or late afternoon for few minutes a day or twice a week.

Appendix-D

Food Group	No. of Servings /day	Portion size and description
Milk and milk products	2-3	1 serving=1cup of milk or 1 cup of yogurt or 1 slice of cheese or 1 cup of kheer or feerni or other milk-based products equivalent to nutrients supplied by 1 cup of milk). 1 cup of whole milk will provide 15 g carbohydrates, 6 g protein, 8 g fat and 150 calories.
Cereals	4-5	1 serving= 2 slice of bread (toast) or 1 chapatti or 1 cup of cooked rice or 1 cup of cereals equivalent to nutrients supplied by 2 slice of bread. One serving of cereal bread=(2 toast x 28 g=56 g) or equivalent amount of other cereals shall provide 30 g carbohydrates, 6 g protein, 0-2 g fat and 160 Kcal.
Vegetables	2-3	1 serving= ½ cup of cooked non-starchy vegetables or ½ cup of vegetables juice/soup or 1 cup of fresh vegetables/salad). One serving of vegetables will provide 5 g carbohydrates, 2 g protein and 25 calories. One serving of starchy vegetables (1 potato (100 g) or maize (1/2 cup) or peas green ½cup) will provide 15 g carbohydrates, 3 g protein, 0-1 g fat and 80 calories.
Fruits	2-3	1 serving=1 medium size banana or 1 apple or 1 peach or 2-3 plums or 3-4 apricots. Each serving will provide 20 g carbohydrates and 80 calories.
Meat and pulses	2-3	1 serving of meat (28 g lean meat=2-3 small pieces of meat or 1-2 pieces of fish or 1 egg, or ½ cup of cooked pulses). One serving of lean meat will provide 7 g protein, 3 g fat and 55 calories. One serving of meat with medium fat will provide 7 g protein, 5 g fat and 75 calories. One serving of meat with high fat will provide 7 g protein, 8 g fat and 100 calories.

Salt, Sugar and Oil

1. Use minimum amount of salt, sugars and oils in cooking, use less than 5 g salt/day, less than 10% calories from free sugars and less than 10% of total fat calories from saturated fat.
2. Avoid junk, refined foods, sweets, soft drinks, French fries, samosa and other deep fried foods.
3. Use clean water for washing vegetables and fruits and protect foods from flies, pathogens, dirt, ° cover and refrigerate them at 5 C or below for a day only.

Sample menu.

Time	Food	Portion Size	Calories (Kcal)	Protein (g)
**Breakfast**	Roti/toast/cereals*	1-2 chapatti	160-320	6-12
	Egg fried‡	1 medium	90	6
	Apple or any other seasonal fruit¶	1 medium	80	----------
	Milk†	1 cup	150	6
**Snack**	Shami sandwich‡‡	1	180	8
**Lunch**	Spinach and potato curry§	1 plate	109	3
	Roti	2 chapatti	320	6-12
	Yogurt†	½ cup	75	3
	Salad§	1 bowl	25	---------
**Snack**	Banana or any other seasonal fruit	1 medium	80	---------
**Dinner**	Pulao**	1 plate	250	8
	Fish ‡	1-2	127-254	10-20
	Salad§	½-1 bowl	13-25	--------
	Raita†	½-1 cup	75-150	3-6
	Apple ¶	1 medium	80	--------
**Snack**	Peanuts***	1 serving	160	7
	Kheer†	1 cup	150	6
		Total	2124-2498 Kcal	72-97 g

*1 roti or 2 toast or ½ paratha or 1 cup fortified cereals providing a similar amount of calories and proteins;
‡ Egg fried or meat, chicken, fish or lentil or chickpea or red beans or shami kabab;
† 1 cup milk or 1 cup kheer or 1 cup yogurt or 1 cup ice cream or 1 slice cheese or any other milk-based product providing a similar amount of calories and proteins;
¶ Banana or apple or any other seasonal fruit providing a similar amount of calories;
‡‡ Shami sandwich or egg sandwich, or chicken sandwich or any other meat or pulses based food providing the similar amount of calories and proteins; §Spinach and potato curry or any other vegetable curry providing a similar amount of calories and proteins
** Pulao or dal-roti or vegetable roti providing a similar amount of calories and proteins;
*** Peanuts or any other nuts providing a similar amount of calories and proteins Note:1 Cup =250 ml; 1 plate pulao or dal or vegetables (100 g cooked food); 1 bowl fresh salad= (100 g); 1 serving peanuts = 1 ounce peanuts

Rafique, I., Saqib, M. A. N., Murad, N., Munir, M. K., Khan, A., Irshad, R., . . . Naz, S. (2020). Adherence to Pakistan dietary guidelines, Findings from major cities of Pakistan. *medRxiv*.
